# Pesticides Drive Stochastic Changes in the Chemoreception and Neurotransmission System of Marine Ectoparasites

**DOI:** 10.3390/ijms17060700

**Published:** 2016-05-31

**Authors:** Gustavo Núñez-Acuña, Sebastián Boltaña, Cristian Gallardo-Escárate

**Affiliations:** Laboratory of Biotechnology and Aquatic Genomics, Interdisciplinary Center for Aquaculture Research, Department of Oceanography, Universidad de Concepción, Concepción 4030000, Chile; gustavonunez@udec.cl (G.N.-A.); sboltana@udec.cl (S.B.)

**Keywords:** *Caligus rogercresseyi*, sea lice, deltamethrin, azamethiphos, glutamatergic synapse, metabotropic receptor, ionotropic receptor

## Abstract

Scientific efforts to elucidate the mechanisms of chemical communication between organisms in marine environments are increasing. This study applied novel molecular technology to outline the effects of two xenobiotic drugs, deltamethrin (DM) and azamethiphos (AZA), on the neurotransmission system of the copepod ectoparasite *Caligus rogercresseyi*. Transcriptome sequencing and bioinformatics analyses were conducted to evaluate treatment effects on the glutamatergic synaptic pathway of the parasite, which is closely related to chemoreception and neurotransmission. After drug treatment with DM or AZA, stochastic mRNA expression patterns of glutamatergic synapse pathway components were observed. Both DM and AZA promoted a down-regulation of the *glutamate-ammonia ligase*, and DM activated a *metabotropic glutamate receptor* that is a suggested inhibitor of neurotransmission. Furthermore, the delousing drugs drove complex rearrangements in the distribution of mapped reads for specific metabotropic glutamate receptor domains. This study introduces a novel methodological approach that produces high-quality results from transcriptomic data. Using this approach, DM and AZA were found to alter the expression of numerous mRNAs tightly linked to the glutamatergic signaling pathway. These data suggest possible new targets for xenobiotic drugs that play key roles in the delousing effects of antiparasitics in sea lice.

## 1. Introduction

Chemical signals and cues constitute the main mechanisms of communication between animals in aquatic environments. Identifying these chemical communications and discriminating between evolved functions (signals) and unintentional releases (cues) are foremost challenges in aquatic biology. Importantly, by fully understanding the recognition systems of chemical communication in marine systems, knowledge regarding biotic interactions could advance more rapidly. Chemical cues play critical roles at every level in marine systems [1,2], but their presence in the environment and recognition by and impact on organisms are not completely understood. For most marine species, chemical cues determine whether they consume, fight with, run from, or mate with the cue-emitting organism, as well as whether they are eaten, infected, or overgrown by natural predators and/or parasites. The molecules involved in these processes are known as allelochemicals (interspecific communication) and pheromones (intraspecific communication) [3]. Besides chemical signals, which are intentionally released by the sender, receivers also react towards unintentionally released chemical cues [4]. While chemical signaling is usually advantageous to the signal-emitting organism, the release of chemical cues usually results in either neutral or damaging effects. In contrast, allelochemicals, such as kairomones, are mostly beneficial to the sender and are often used in predacious or parasitic relationships [5].

In marine ectoparasites, allelochemicals facilitate significant biological processes such as kin recognition, foraging, host infection, and mate pairing [6,7]. Some kairomones that mediate host recognition in marine ectoparasites have been identified [8,9], but most evidence is limited to the Northern Hemisphere ectoparasite *Lepeophtheirus salmonis*. Regarding marine ectoparasites from the Southern Hemisphere, host recognition processes mediated by semiochemicals have been suggested for the sea louse *Caligus rogercresseyi* [10]. Notwithstanding these contributions in host recognition, the chemoreception process of marine parasitic species remains unclear, especially at a molecular level.

The chemosensory system in animals includes key downstream pathways mainly related to the recognition of chemical stimuli and transduction of neurotransmission signals, including of glutamate receptors, adrenergic receptors, and acetylcholine. Related to this, G protein-coupled receptors (GPCRs) represent the majority of membrane-bound proteins with known functions in signal neurotransmission and sensory perception [11,12]. Within the GPCRs, metabotropic glutamate receptors (mGluRs), play key roles in signal transduction, especially in fast synaptic transmission, such as in the glutamatergic synapse pathway [13]. In mammals, mGluRs include a family of proteins with seven transmembrane domains that are subdivided into three groups containing at least eight different proteins, all of which couple to G proteins [14]. In turn, ionotropic glutamate receptors (iGluRs) correspond to a group of ion channels with affinity for a diverse array of ligands [15]. Various members of the iGluR family have been described in insect species, and a subfamily of ionotropic receptors (named IRs) can bind different ligands, including some volatile chemicals [16]. Additionally, these IRs share structural amino acid components with iGluRs and have similar gene expression patterns as insect odorant receptors, suggesting a role in chemical communication [17,18]. Recently, a group of iGluRs was identified in *C. rogercresseyi*, thus indicating a potential role of this group in the chemosensory reception system [19]. However, the downstream signaling pathway of the chemosensory system has not been identified in sea lice. Furthermore, while some glutamate receptors are characterized as drugs targets [14,20], it is not clear whether delousing drugs are also able to trigger expressional changes in mGluRs and iGluRs. In this context, there is just a few studies evaluating the effect of these drugs to ectoparasite abilities to recognize hosts, and they exhibited contradictory results. In the bug *Trissolcus basalis*, an alteration of the host recognition behavior of the parasite after exposure to sublethal concentrations of deltamethrin has been reported [21]. In contrast, in the egg parasitoid *Anagrus nilaparvatae* there were no alterations in the host recognition mechanism after exposure to sublethal concentrations of the same drug [22]. Besides, there are no studies describing any behavior related to host identification in marine ectoparasites.

The marine ectoparasite *C. rogercresseyi* was used as a model in the current study. This copepod causes major economic losses to Chilean salmon farms [23] by inducing caligidosis, a disease mainly treated with delousing drugs such as pyrethroids and organophosphates [24]. The main focus of this study was to infer the effects of xenobiotic drugs on glutamatergic synapse-related genes in *C. rogercresseyi* through *in silico* analyses in transcriptomes exposed to deltamethrin (DM, a pyrethroid) or azamethiphos (AZA, an organophosphate). The methodological approach was based on a deep analysis of transcriptional performance (RNA-seq), which allowed the determination of the effects of the drugs at different transcriptomic levels by estimating total mRNA abundance, candidate gene transcription, transcriptional directional shifts, and mapping arrangement. The present study is innovative in that it evaluates a novel gene-signaling pathway in marine copepods while under antiparasitic drug treatment and incorporates new concepts derived from an *in silico* strategy.

## 2. Results

### 2.1. Transcriptomic Responses of Glutamatergic Pathway Components to Antiparasitic Treatment: A Global Comparison

In the marine environment, information remains lacking to explain the underlying molecular basis and signal perception of the chemosensory system, as well as the effects that chemicals or drugs would have on it. Therefore, having established the existence of a chemosensory mechanism through the glutamatergic pathway, transcriptome analyses were conducted on adult sea lice exposed to either DM or AZA, both chemical antiparasitics used in the control of *C. rogercresseyi* in salmon farming. Through this, a core set of transcripts tightly linked to the glutamatergic synapse pathway was identified in response to DM and AZA.

**Figure 1:**
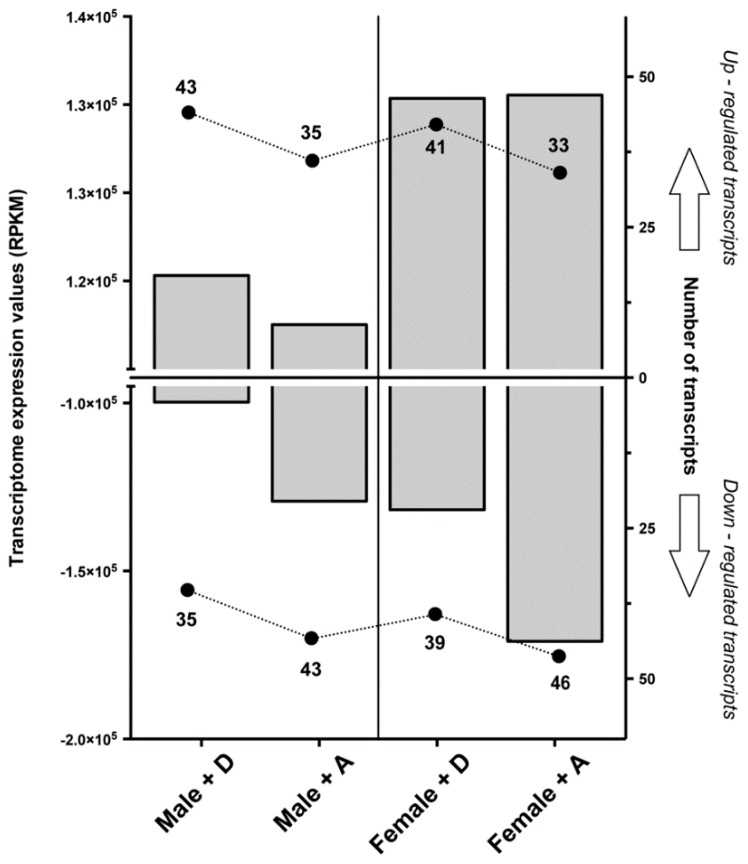
Total transcriptomic response of the glutamatergic synapse pathway in male and female *Caligus rogercresseyi* exposed to xenobiotic drugs. In the left *Y*-axis (gray bars), the sum of expression values for up- and down-regulated transcripts after drug exposure are shown. Values correspond to differences in Reads Per Kilobase of transcript per Million mapped reads (RPKM) values as compared to the same transcripts in the control condition. In the right *Y*-axis (plot points), the total number of up- and down-regulated transcripts after drug application are shown. +D: Deltamethrin, +A: Azamethiphos.

The abundances of glutamatergic synapse pathway transcripts derived under DM and AZA treatment were significantly different in regards to the total number of differentially expressed transcripts and intensity (fold change > |−2|). In total, 176 transcripts were differentially expressed (Reads Per Kilobase of transcript per Million mapped reads, RPKM) between the treatment groups (88 DM and 88 AZA) and the control. Peak intensity was observed with AZA (Δ total RPKM of 243,241 in 88 transcripts as compared to the control, *versus* 230,106 for DM). The expression profiles obtained for glutamatergic synapse-related genes highlighted a marked contrast between sexes during the parasitic drug response. Specifically, a greater number and higher intensity (fold change) of induced transcripts were observed in female sea lice than in male specimens (Figure 1).

**Figure 2:**
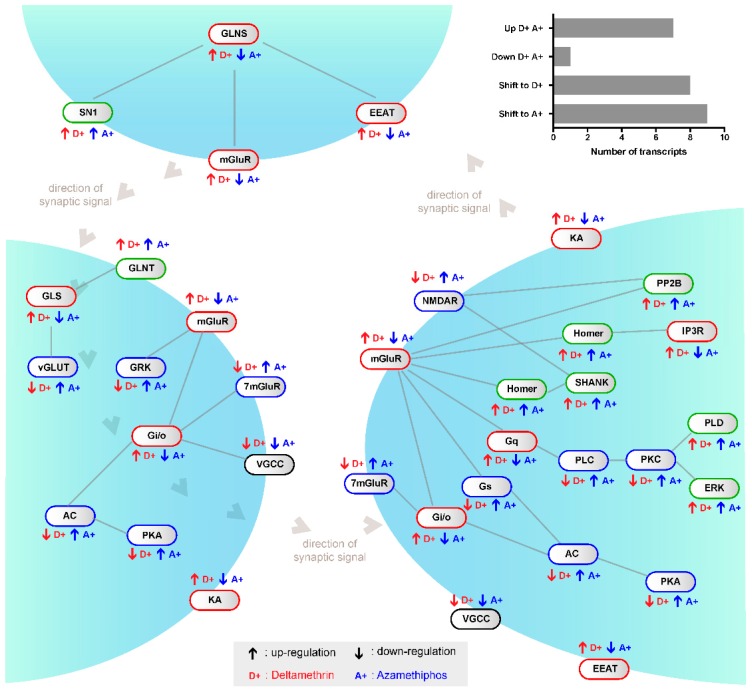
Modulation of glutamatergic synapse pathway transcription levels by xenobiotic drugs in *Caligus rogercresseyi*. Abbreviations are per established GeneCards nomenclature. Green border: genes up-regulated by both drugs; black border: genes down-regulated by both drugs; red border: genes up-regulated by deltamethrin but down-regulated by azamethiphos (*i.e.*, shift to deltamethrin); and blue border: genes down-regulated by deltamethrin but up-regulated by azamethiphos (*i.e.*, shift to azamethiphos).

**Figure 3:**
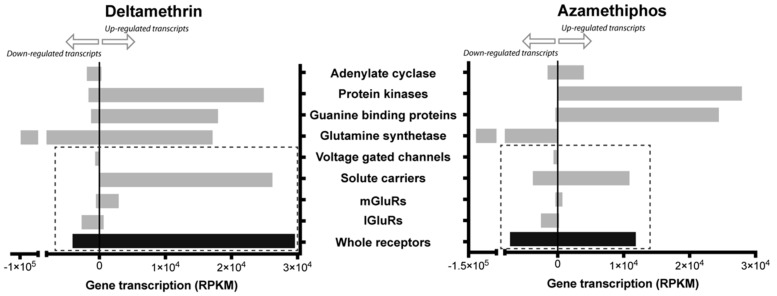
Expression levels of glutamatergic synapse-related genes grouped by molecular functions. Gene expression was measured as the sum of total RPKM after exposure to antiparasitic drugs as compared to control samples. Gray bars correspond to group of contigs clustered by gene family; black bar corresponds to the sum of all the groups that are related to receptors gene families. Dotted rectangle includes the gene families related to membrane receptors.

To determine whether delousing drugs simply shifted the global gene expression profile of the glutamatergic synapse-related transcripts, the directional shift of the regulated transcripts was measured within the pathway; in other words, up-regulation with one drug but down-regulation with the other drug was identified. Significant differences were recorded between AZA and DM in regards to gene shift, where eight shifts were found with DM, nine shifts with AZA, and eight genes with no shifts (Figure 2). Non-shifted transcripts corresponded to the down-regulated voltage-dependent calcium channel and to up-regulated genes by both drugs, including solute carrier family 8, members 3 (SN1) and 2 (GLS); protein phosphatase (PP2B); mitogen-activated protein kinase 1; phospholipase D; and the mGluR regulators ankyrin repeat domain (SHANK) and Homer. Regarding other receptor proteins, solute carrier family 1, member 2 (EEAT); ionotropic kainate receptors (KA); and mGluR 3–5 were shifted to DM, while the 7-transmembrane glutamate receptor domain containing protein (7mGluR) and ionotropic NMDAR receptors were shifted to AZA. Additionally, the G protein O and I subunits were shifted to DM, whereas the S unit was shifted to AZA (Figure 2). Although both drugs notably down-regulated glutamine synthetase, also known as glutamate-ammonia ligase (GLNS), some contigs related to this gene were up-regulated by DM. Furthermore, receptor activity was more closely associated with DM, especially for the mGluRs and solute carriers (Figure 3). Therefore, the different mRNA abundances were not a consequence of a random shift. Rather, these differences reflect drug-dependent changes in transcript abundance as part of the chemosensory response, suggesting an increased focus on transcription. This coordinated response indicates that delousing drugs can drive directionally juxtaposed variations in the mRNA abundances of the glutamatergic pathway.

The global expression profiles of sea lice exposed to both DM and AZA were also assessed. A total of 119 transcripts scored positive for reliable annotation among all samples and were tightly linked to chemosensory components. These transcripts were used as statistical analyses that determined significantly different mRNA abundances between drug treatments. Principal components analysis (PCA) and hierarchical clustering were used to determine the contribution that each mRNA had in the response to DM and AZA treatments. Particularly, the DeDaL-PCA Cytoscape plugin combines classical and advanced data dimension reduction methods with the algorithms of network layout inside the Cytoscape environment. The genetic interactions between these genes and the epistatic profiles (computed only for this group of genes) were selected from de global expression profile (RNA-seq). Definitions for the chemosensory pathway were taken from the KEGG database.

**Figure 4:**
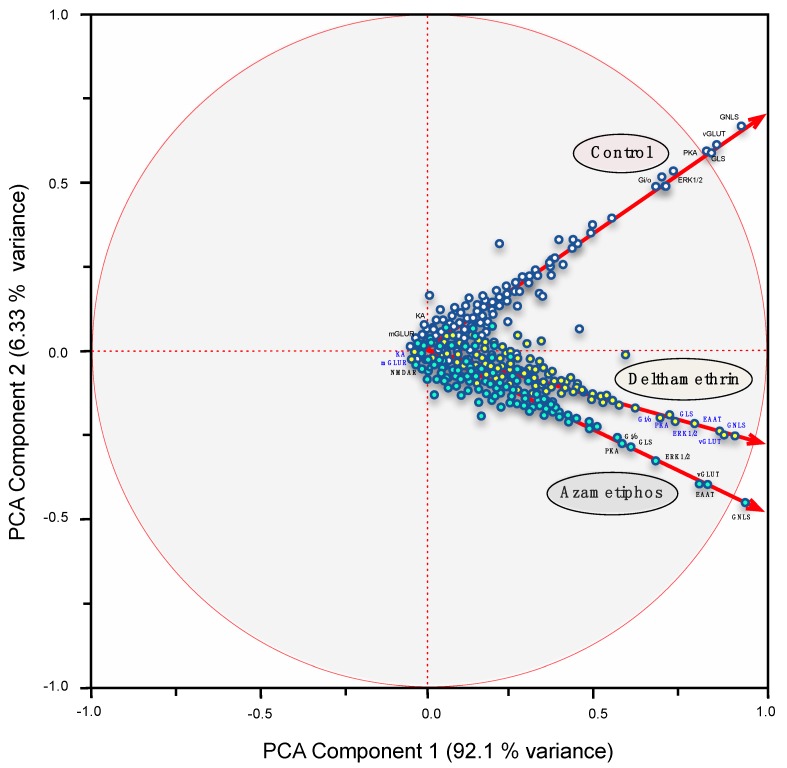
The DeDaL-PCA Cytoscape plugin was used to visualize the network of genetic interactions for downstream components of the glutamatergic pathway affected by delousing drugs. Different node colors indicate distinct mRNA regulation after drug exposures. A principal components analysis (PCA) was applied to the network-smoothed profile. DeDaL used the elastic map (elmap) algorithm for computing non-linear principal manifolds. A factorial map of the PCA was applied to data for components of the glutamatergic pathway, which are represented as colored circles for each treatment condition (white: control; yellow: deltamethrin; and blue: azamethiphos). The portion of the variance explained by the principal component is indicated in parentheses.

Differences were obtained between the standard and DeDaL-PCA-based organic layouts for this network of genetic interactions (Figure 4). The DeDaL-PCA was computed without applying data matrix double-centering to take into account tendencies of genes to interact with a smaller or larger number of other genes, thereby estimating the effects of AZA or DM on the mRNA abundances of chemosensory components. Both PCAs clearly highlighted differences between the treatments and against the control group (Figure 4). In the standard PCA, the local glutamate receptors genes GLNS, EEAT, SN1, and GLS were distinctly positioned by DM and AZA treatments, a result similarly obtained by the DeDaL-PCA. Additionally, some genes, such as KA and mGluR, showed weak expression patterns in both treatments. For both treatments, the two principal factors together explained close to 99% of expressional variability. This suggests that both delousing treatments had a similar effect on the expression profile of chemosensory components.

### 2.2. mGluR Characterization in Caligus Rogercresseyi

Two complete mRNA sequences were obtained for mGluRs in C. rogercresseyi. These sequences were termed Cr-mGluR-A and Cr-mGluR-B (GenBank Accession Numbers KT599917 and KT599918, respectively). BlastX analysis found Cr-mGluR-A similar to the mGluR3-like gene in diverse arthropod species (*E*-value = 0) such as Microplitis demolitor, Nasonia vitripennis, and Megachile rotundata. In turn, Cr-mGluR-B was similar to mGluR in Tribolium castaneum (*E*-value = 7.42 × 10^−68^) and mGluR3 in Strongylocentrotus purpuratus (*E*-value = 1.96 × 10^−65^).

**Figure 5:**
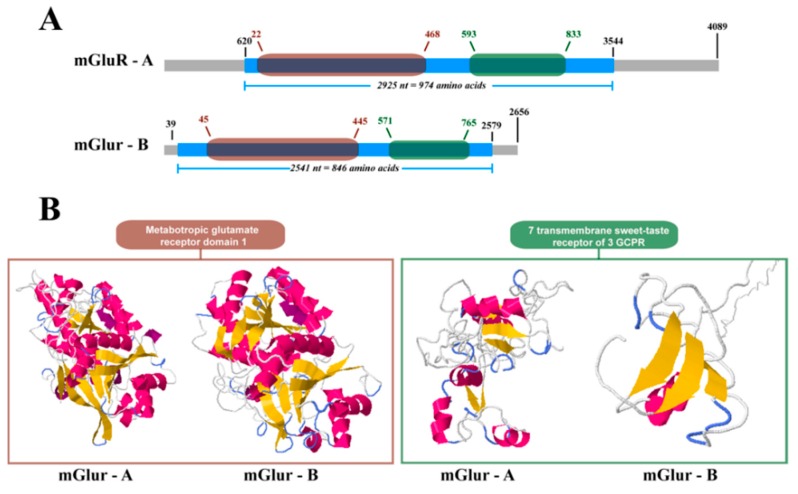
Structural characteristics of two metabotropic glutamate receptor (mGluR) sequences in *Caligus rogercresseyi*. (**A**) Sequence schemes for Cr-mGluR-A and Cr-mGluR-B, indicating the position of the open reading frame (blue rectangle), metabotropic glutamate receptor domain 1 (brown rounded-rectangle), and 7 transmembrane sweet-taste receptor of 3 GPCR (green rounded-rectangle); (**B**) Predictive model of both domains in each mGluR sequence. Brown and green lines correspond to the same domains described in **A**, respectively. In the models, yellow arrows correspond to beta sheets and pink regions to alpha helix.

Both sequences exhibited coding sequences and the 3′ and 5′ untranslated regions. The complete sequence, coding sequences, and untranslated regions of Cr-mGluR-A were larger than Cr-mGluR-B (Figure 5A,B). In both Cr-mGluRs, nucleotide regions homologous to the mGluR domain 1 and to the 7-transmembrane sweet-taste receptor of 3 GPCR domain were found (Figure 5A). Structural differences were assessed with the PSIPRED v3.3 and DomSerf v2.0 tools, which showed the crystal forms of the Cr-mGluRs and provided information on the structural disposition of both proteins. The *in silico* crystallized structures of Cr-mGluR-A and Cr-mGluR-B assumed active conformations with well-defined canonical dispositions of α-helixes and β-sheets in the first domain, but with dissimilar fragments in the second domain (Figure 5B). Finally, the conformations adopted by the α-helixes and β-sheets differed from those seen in the mGluR structures of other organisms.

### 2.3. Gene Transcription of Cr-mGluR after Drug Treatments and Mapping Arrangement Analysis

**Figure 6:**
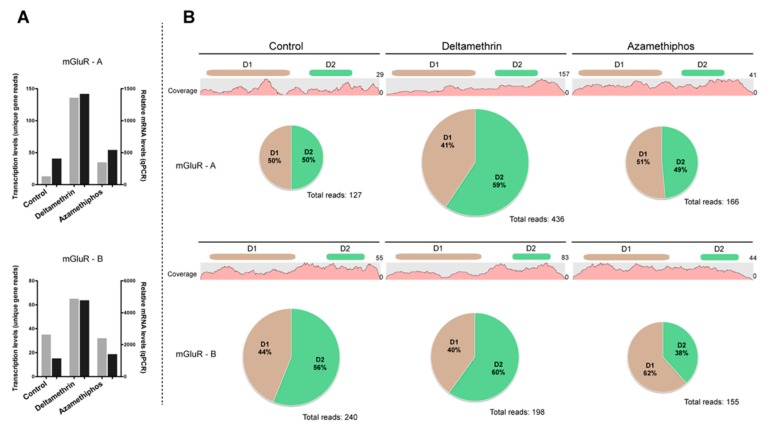
Expression levels of two mGluR genes in *Caligus rogercresseyi* treated with delousing drugs. (**A**) Validation of gene transcription levels by qPCR analyses. Gray bars correspond to expression changes obtained by *in silico* analysis, and black bars expression levels inferred by qPCR reactions; (**B**) Distribution of mapped reads in both mGluR transcripts after exposure to deltamethrin or azamethiphos. D1: metabotropic glutamate receptor domain 1; and D2: 7 transmembrane sweet-taste receptor of 3 GPCR.

The gene transcriptions of Cr-mGluR-A and Cr-mGluR-B were evaluated through RT-qPCR and *in silico* (RPKM values) analyses (Figure 6A). Both transcripts were activated by DM exposure, showing expression levels 2-fold higher than control samples. In contrast, gene transcription levels did not vary following AZA treatment.

To determine the impact of DM and AZA on the mapping arrangement of Cr-mGluR-A and Cr-mGluR-B, the distribution of reads in each domain was checked (Figure 6B). For Cr-mGluR-A, the control samples exhibited an equal distribution of mapped reads for both the mGluR 1 and the 7-transmembrane sweet-taste receptor of 3 GPCR domains. Exposure to DM resulted in a redistribution of mapped Cr-mGluR-A reads, with a 9% increase in the total number of reads mapped in the mGluR domain 1. However, AZA treatment did not result in variations to the mapping arrangement of Cr-mGluR-A as compared to the control. Regarding Cr-mGluR-B, mapping arrangement, as compared to the control, varied by domain, with 44% of mapped reads in the mGluR domain 1% and 56% in the 7-transmembrane sweet-taste receptor of 3 GPCR. Additionally, DM triggered a 4% increase in the total number of mapped reads for the 7-transmembrane sweet-taste receptor of 3 GPCR in Cr-mGluR-B, but AZA exhibited an opposite trend, increasing the mapped reads of the mGluR domain 1 by 18%.

## 3. Discussion

Chemical cues and signals are involved in every aspect of the copepod life-cycle, including in finding and assessing mates, determining and stabilizing dominance hierarchies, finding food, recognizing kin, foraging, and infecting a host [1]. However, characterizing chemical communication, recognition, and responses in the aquatic environment are challenging due to small chemical amounts and a complex background of other compounds. Cues and signals are transduced from the sense organs into sparse and stimulus-specific activity patterns across large populations of perception cells [25,26]. Nevertheless, the subsequent processing steps are poorly understood.

The complexity of the receptor recognition pattern could be driven by several factors (*i.e.*, size, landscape, and noise of chemical signal). Using the expressional pattern of the downstream glutamatergic pathway, a spatial *in silico* pattern of neuronal synaptic activation was constructed. Glutamate signaling includes mGluRs and iGluRs [13], both of which are implicated in the recognition of chemical communications. The impact that delousing drugs had on the *C. rogercresseyi* transcriptome was systematically assessed, revealing significant remodelling of the transcriptional profile, with particular divergence in the response between sexes. This sex-dependent finding is in agreement with previous results in *C. rogercresseyi* [27,28] and highlights the effects that DM and AZA can have on the magnitude of the transcriptomic response. First, in both cited studies most of the survivors were female individuals. Furthermore, both studies described a wide number of differentially expressed genes in both sexes, including more abundance of female-exclusive transcripts in the delthamethrin treatment. Besides, both drugs changed the expression pattern of different genes of the glutamatergic synapse pathway. As there were no other known variables, transcriptomic profile differences were assumed to be solely due to the chemical composition of the different drugs and, additionally, to male/female variations in drug recognition. This assumption was supported by variations in transcript numbers and intensity (Figure 1).

Comparisons including pathway-drug interactions indicated different treatment responses in regards to magnitude and intensity. These variations were identified at multiple levels, including for the expression of single mRNAs, the regulation of mRNA expression, and for the directional juxtaposition of shifts linked to increased variations in mRNA abundance, signifying a change in functional output (excited or inhibited, Figure 2) [11]. The obtained results suggest that DM promotes significant changes in specific Gi/Go subunits of the G protein that could drive the inhibition of glutamatergic synapse transmission [29,30,31]. This regulation might affect drug effectivity in relation to parasite survival. Specifically, DM rapidly killed both males (73%) and females (80%), while AZA killed only 60% of males and 46.7% of females. Supporting this observation on glutamatergic neurotransmission, both antiparasitics also promoted significant consequences in other nervous system elements, such as NOTCH and the ABC transporters [32]. There is increasing evidence that delousing drugs are able to control unknown regulatory elements of the nervous system, consequently indicating a close interaction between drugs and hypothetical drug resistance [33]. In this context, the present study expands on the description of potential targets for DM an AZA, which was previously limited to specific genes, such as the acetylcholinesterase enzyme in the case of AZA [34].

In support of this, a significant effect of both drugs on GLNS mRNA transcript abundance was significantly affected by both DM and AZA (GLNS, Figure 3 and Figure 4). As a multi-functional enzyme tightly related to drug-resistant phenotypes [35], GLNS catalyses the conversion of glutamate and ammonia into glutamine, which plays a key role in the activation of GPCRs, thus promoting glutamatergic synapse transmission [36]. In the present analysis, the importance of GLNS down-regulation could be in the lethality effect of xenobiotic drugs in this marine ectoparasite. Additionally, GLNS activity is pivotal to olfactory perception in other arthropods such as *Drosophila melanogaster* [37]. Therefore, the present results suggest that DM and AZA may also inhibit olfactory perception in *C. rogercresseyi*.

A notable effect on the neurotransmission and olfactory systems was triggered in *C. rogercresseyi* following xenobiotic drug exposure. Integrative computational and analytical assessments of transcriptome data [38] further support this observation. Other *in silico* approaches, such as those focused only on mRNA fragments, could be biased since gene expression measures are extrapolated from short sequences [39]. Full-transcript *in silico* analyses allow for inferring the presence of transcript variants and isoforms due to overlapping mRNA sequences [40]. The novel method applied in the present study was based not on developing an automatic tool to infer transcriptional changes at the deepest level, but rather on monitoring specific mRNA sequence mappings to detect differences in the read distribution arrangement of specific sequences as a result of drug exposure. For this, the mapping arrangement of Cr-mGluRs were assessed first due to their importance in signal transduction of the glutamatergic synapse [13]. To prevent incorrect interpretations of results, technical biases have to be discarded through strong bioinformatics approaches that incorporate the assembly of reads into contigs [41]. This computational and analytical method also discards other bias sources from the library construction procedure, which, in the case of Illumina sequencing, would include the use of random hexamers, selective adapter ligation, and a biased PCR system [42].

The observed mapping rearrangements might explain the biological effect of DM and AZA on the polymerase efficiency and transcriptional performance of the selected mRNAs. This was also observed in untreated individuals at different developmental stages (Figure S1). Detailed mapping analyses exhibited greater read depth at the 3′ untranslated region of transcripts, especially in samples exposed to DM (Supplementary Figures S2 and S3). The poly-A tails located at the end of 3′ untranslated regions allows subtractive hybridization to eliminate non-messenger RNA in the library construction process [42]. A technical limitation of RNA-seq analysis is that it can only display a time-dependent snapshot of the transcriptome. Therefore, short mRNA fragments present in the cytoplasm may also be retained as poly-A tails could attach to these sequences. This observation suggests that DM may generate primary and secondary effects on mRNAs transcription, and, in some cases, cell machinery appears to respond by activating post-transcriptional regulations. However, further analysis should be carried out to confirm this hypothesis. The present analyses highlight that the key functions of the sensorial and nervous systems are inhibited in distinct transcriptional phases that are strongly triggered by the delousing drugs DM and AZA. Although the deleterious effects of xenobiotics are somewhat defined in other marine parasites [43], the present results and methodology expand previous observations.

## 4. Materials and Methods

### 4.1. Bioassays and Transcriptome Sequencing

Sea lice (*Caligus rogercresseyi*) were cultured under controlled laboratory conditions according to the protocol described by Bravo *et al.* (2010) [44]. After obtaining active male and female adults, bioassays were performed with the delousing drugs deltamethrin (AlphaMax™, PHARMAQ AS Chile Ltda., Puerto Montt, Chile) and azamethiphos (Byelice^®^, Bayer S.A. Chile, Santiago, Chile).

Bioassays and transcriptome sequencing were performed for DM according to Chavez-Mardones *et al.* (2014) [45] and for AZA according to Valenzuela-Muñoz *et al.* (2015) [28]. Briefly, adult male and female sea lice were incubated for 30 min in either DM (2 ppb) or AZA (3 ppb) and then transferred to a drug-free medium. Drug concentrations were selected according to previously obtained results [26,43]. Control sea lice were maintained under the same culture conditions as experimental sea lice but were not exposed to any drug. At 24 h post-treatment, survival rate and the number of immobilized lice were obtained for each assay. Surviving lice were transferred to cryogenic tubes containing RNAlater^®^ (Ambion^®^, Thermo Fisher Scientific^®^, Waltham, MA, USA), fixed, and stored at −80 °C until molecular analyses. Sea lice RNA was extracted using the RiboPure RNA Isolation Kit™ (Ambion^®^ Thermo Fisher Scientific^®^, Waltham, MA, USA), and cDNA libraries were constructed using the TruSeq RNA Sample Preparation v2 Kit (Illumina^®^, San Diego, CA, USA). High-throughput sequencing was conducted with an Illumina MiSeq™ System (Illumina^®^, San Diego, CA, USA) Male and female samples were separately sequenced for each group, and each group was sequenced twice (*i.e.*, two technical replicates).

### 4.2. In Silico Analyses of Glutamatergic Synapse Pathway in Sea Lice to Antiparasitic Drugs

Millions of short sequences obtained from Illumina sequencing runs for each assay were trimmed, filtered, and assembled into contigs following the methodology established in a transcriptomic characterization of *C. rogercresseyi* [46]. For MultiBlast analysis, sequences of glutamatergic synapse pathway genes from other arthropod species were used as references for identifying these genes in the *C. rogercresseyi* transcriptome. *In silico* analyses were performed using the CLC Genomic Workbench software (version 8.0, CLCBio^®^, Aarhus, Denmark). MultiBlast sequence results with *E*-values higher than 1 × 10^−20^ were discarded. In cases where two or more sequences were related to the same gene, the sequences were aligned with the corresponding reference, and the sequence with the highest coverage and homology was selected. The selected contigs were used as new specific references for evaluating corresponding expression levels through the CLC Genomic Workbench RNA-seq module. The RPKM value was calculated for each contig in the different experimental groups. With this dataset, the total transcriptomic expression of the assessed genes was obtained by applying logic functions according to individual transcript expression patterns in a Microsoft Excel spreadsheet (Table S1). These analyses provided a count of the up- and down-regulated transcripts, as well as an account of respective total expressional changes.

Transcriptional directional shifts of genes from the glutamatergic synapse pathway were evaluated using the methodological approach described by Boltaña *et al.* (2013) [47]. A directional shift is defined as a variation in the transcriptional direction (up-regulation, down-regulation) of a single gene under different conditions. Directional shifts were classified within the obtained transcriptional data after exposure to DM or AZA, with variations inferred as compared to the basal expression levels of the control group.

### 4.3. Statistical Analyses

Hierarchical clustering analysis was performed using the Euclidean distance matrix and complete linkage method. Principal component analysis of gene ontology terms showed two-dimensional views, as retrieved from the DeDaL plugin of the Cytoscape environment [48]. This analysis was used to visualize the relatedness of all RNA-seq samples. All *p*-values were adjusted with a false discovery rate correction for multiple testing by the Benjamini-Hochberg method [49]. All genes with false discovery rate-corrected *p*-values <0.05 were considered significant. The expression of genes found to be significantly different between DM and AZA treatments were further characterized by hierarchical clustering analysis. Hierarchical clustering was based on the expression pattern across the sampled population, thereby identifying gene clusters with common expression profiles. Sample variances were homogeneous (normal distribution). Principal components analysis in DeDaL was computed using singular value decomposition, as described by Gorban and Zinovyev (2009) [50]. This allowed using missing data values without pre-imputing the values, but data points containing more than 20% of missing values were filtered out from the analysis. DeDaL computes the first ten principal components if there are more than ten data points. DeDAL also computes the *k* principal components if there is *k* + 1 data points, *k* < 10. After computing the principal components, DeDaL reports the amount of variance explained by each of the principal components. DeDaL is the first plugin able to construct biological network layouts from high-throughput data in the Cytoscape environment.

### 4.4. Structural Analysis of Cr-mGluRs

From the filtered contigs, transcripts annotating for the metabotropic glutamate receptor were extracted. Six contigs were found and assessed through multiple alignments using the CLC Genomic Workbench software to infer if they could correspond to different transcripts. Two of these transcripts were selected as they encompassed the complete mRNA sequence, including coding sequences (open reading frame) and untranslated regions. These two sequences were termed Cr-mGluR-A and Cr-mGluR-B and were deposited in the NCBI GenBank Database (Accession numbers KT599917 and KT599918).

Protein domains and the 3D structural models of Cr-mGluR-A and Cr-mGluR-B were predicted with the online tool PSIPRED [51]. Specifically, the PSIPRED v3.3 and DomSerf v2.0 tools were used to predict the secondary structure of proteins and model the domains, respectively.

### 4.5. Gene Transcription and Imbalance Analysis of mGluR

Gene transcription levels (*i.e.*, mRNA abundance) of Cr-mGluR-A and Cr-mGluR-B were evaluated by calculating RPKM values. These values were validated through quantitative PCR runs using specifically designed primers. The qPCR runs were performed in a StepOnePlus™ qPCR System (Applied Biosystems^®^, Foster City, CA, USA) using the ΔΔ*C*_t_ quantification method. Reactions were conducted using the Maxima SYBR Green qPCR Master Mix™ (Thermo Scientific^®^, Thermo Fisher Scientific^®^, Waltham, MA, USA) following the manufacturer’s protocol. Data were normalized against the expression levels of β*-tubulin*, which was previously validated as an endogenous control for this species [46].

Furthermore, a novel approach was used to infer the transcriptional changes of the Cr-mGluR-A and Cr-mGluR-B sequences resulting from exposure to DM or AZA. Specifically, the distributions of the mapped reads were evaluated in both Cr-mGluR transcripts. For each transcript, the nucleotide sequence homologous to each specific domain (abbreviated as D1 and D2) was extracted to separately map all of the reads obtained for each treatment (control, DM, and AZA). The mapped reads corresponding to each domain were counted for both Cr-mGluRs and for each treatment. Then, the percentage of reads mapped to domains was determined. To prevent bioinformatics bias, the extracted sequences were compared and then blasted against the complete database of *C. rogercresseyi* transcriptomes to determine fragment uniqueness.

## 5. Conclusions

Overall, both xenobiotic drugs caused notable changes in the expression patterns of glutamatergic synapse pathway components, leading to stochastic disorder in the mRNA abundances for most of the regulatory transcripts important to this pathway. Among these essential genes, metabotropic receptors and glutamate-ammonia ligase are considered key elements in understanding the effects of pesticides on marine ectoparasites. Forthcoming research should study transcriptome regulation of transcriptomes exposed to drugs, using a fine-tuning approach, such as the mapping arrangement.

## Supplementary Materials

Supplementary materials can be found at http://www.mdpi.com/1422-0067/17/6/700/s1.

## Abbreviations

DM	Deltamethrin
AZA	Azamethiphos
mRNA	messenger RNA
GPCRs	G protein-coupled receptors
mGluR	metabotropic receptor
iGluR	ionotropic glutamate receptor
IR	ionotropic receptor
RPKM	reads per kilobase of transcript per million mapped reads
SN1	solute carrier family 8, member 3
GLS	glutaminase (solute carrier family 8, member 2)
PP2B	protein phosphatase 2B
EEAT	solute carrier family 1, member 2
KA	ionotropic kainate receptor
7mGLuR	7-transmembranse glutamate receptor domain containing protein
GLNS	glutamine synthetase/glutamate-ammonia ligase
PCA	principal component analysis
KEGG	Kyoto Encyclopedia of Genomes and Genes
