# Supplementary material for: Pesticides Drive Stochastic Changes in the Chemoreception and Neurotransmission System of Marine Ectoparasites
Source: Int J Mol Sci. 2016 May 31;17(6):700. doi: 10.3390/ijms17060700 (PMC4926324; doi:10.3390/ijms17060700)
Supplement: Supplementary file 1 [file ijms-17-00700-s001.pdf]

Supplementary Materials: Drive Stochastic Changes in the Chemoreception and Neurotransmission System of Marine Ectoparasites

Gustavo Núñez-Acuña, Sebastián Boltaña and Cristian Gallardo-Escárate

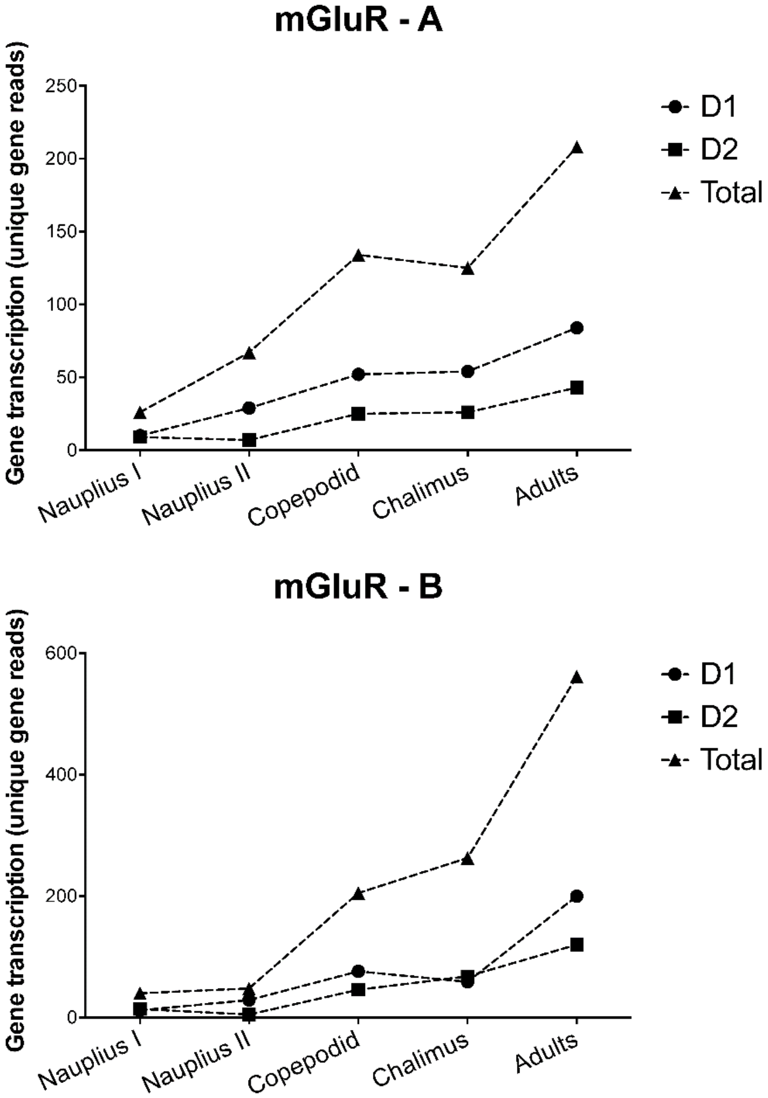

Figure S1. Gene transcription by domains of mGluR-A and mGluR-B in lifecycle stages of *Caligus rogercresseyi*. (D1: metabotropic glutamate receptor domain 1; D2: 7 transmembranse sweet-yaste receptor of 3 GPCR).

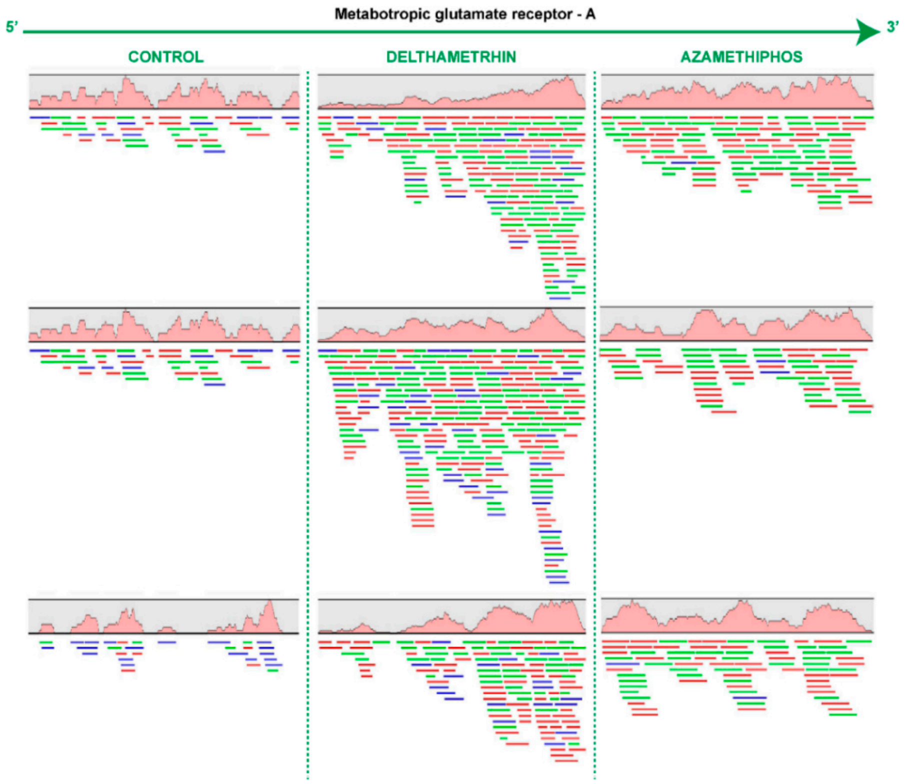

Figure S2. Arrangement of mGluR-A mapped reads separately by replicates. The direction of all the mappings is 5′ to 3′. Mappings were constructed on CLC Genomic Workbench. Green lines correspond to single reads in (+) direction, red lines to single reads in (−) direction, and blue lines to paired reads.

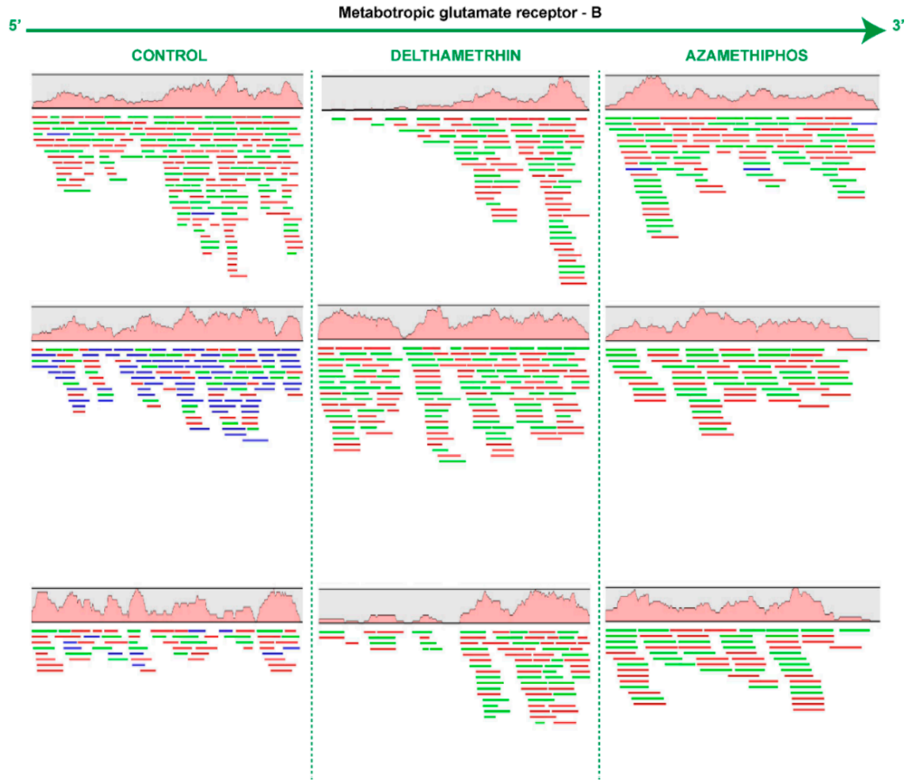

Figure S3. Arrangement of mGluR-B mapped reads separately by replicates. The direction of all the mappings is 5′ to 3′. Mappings were constructed on CLC Genomic Workbench. Green lines correspond to single reads in (+) direction, red lines to single reads in (−) direction, and blue lines to paired reads.

Table S1. List of genes affected by DM and AZA drugs on sea lice.

Contig Number	Blast Result	Male	Male + DM	Male + AZA	Female	Female + DM	Female + AZA
Contig_10248	SH3 and multiple ankyrin repeat domains protein 1-like, partial [Astyanax mexicanus]	3.09825	4.68147	9.59544	4.01297	3.85568	13.07020
Contig_10695	Hypothetical protein DAPPUDRAFT_310221 [Daphnia pulex]	45,109	67,367	70,684	62,706	24,852	1.34793
Contig_11029	Inositol 1,4,5-trisphosphate receptor isoform X4 [Apis mellifera]	23,292	32,109	6738	65,752	59,226	5.13969
Contig_11695	Metabotropic glutamate receptor 3-like isoform X3 [Microplitis demolitor]	42,296	2.10554	1.65690	82,313	3.26233	0
Contig_12773	Adenylate cyclase type 2 [Harpegnathos saltator]	2.54602	1.23927	1.77311	1.54152	1.62089	0
Contig_12789	Hypothetical protein TcasGA2_TC005176 [Tribolium castaneum]	75,221	54,921	94,295	2.23070	2.43126	2.39757
Contig_13144	Hypothetical protein DAPPUDRAFT_319998 [Daphnia pulex]	46,491	0	1.74840	46,532	1.22946	0
Contig_13170	Glutaminase kidney isoform, mitochondrial isoform X3 [Tribolium castaneum]	2.10092	3.13759	3.95048	2.80366	5.55589	0
Contig_13171	Glutaminase kidney isoform, mitochondrial [Cerapachys biroi]	40,258	0	0	0	0	0
Contig_13172	Glutaminase kidney isoform, mitochondrial-like isoform X5 [Musca domestica]	5.75056	5.36755	4.50545	4.49651	5.54434	0
Contig_13277	Calcineurin subunit B isoform 2 [Lepeophtheirus salmonis]	28,483	0	0	23,756	94,154	0
Contig_13402	Glutamate [NMDA] receptor subunit epsilon-2 [Cerapachys biroi]	1005	10,006	0	5588	44,297	0
Contig_13520	Ionotropic glutamate kainate receptor 2-like b [Caligus rogercresseyi]	9.49704	4.94045	6.47960	2.29929	4.19189	3.45980
Contig_13953	Hypothetical protein D910_04195 [Dendroctonus ponderosae]	34,712	20,736	1.30541	34,742	0	0
Contig_1427	SH3 and multiple ankyrin repeat domains protein 3 isoform X4 [Tribolium castaneum]	1.51011	1.59518	3.80914	1.93535	4.13958	8.58465
Contig_14446	Metabotropic receptor [Drosophila sechellia]	9583	14,311	0	15,985	15,838	0
Contig_1526	Mitogen-activated protein kinase 1 [Acyrthosiphon pisum]	8.69124	23.02503	13.50040	26.28538	47.21703	35.22992
Contig_15633	Hypothetical protein TcasGA2_TC004727 [Tribolium castaneum]	1.87208	2.10701	1.53051	1.04095	2.51124	1.94577
Contig_15662	Metabotropic glutamate receptor-like [Metaseiulus occidentalis]	0	0	0	0	0	0
Contig_15714	Hypothetical protein YQE_02510 partial [Dendroctonus ponderosae]	7.07999	5.23441	6.59053	3.62491	3.24409	0
Contig_15768	Protein kinase C, brain isozyme [Cerapachys biroi]	3.01898	2.54837	4.31926	5.91181	4.33896	4.70670
Contig_1592	GK21608 [Drosophila willistoni]	15.36901	29.16631	21.28851	8.59294	15.90553	7.10442
Contig_16122	1-phosphatidylinositol 4,5-bisphosphate phosphodiesterase beta-1-like [Metaseiulus occidentalis]	1.01425	34,764	0	55,472	54,963	1.19242
Contig_16741	1-phosphatidylinositol 4,5-bisphosphate phosphodiesterase beta-1-like [Takifugu rubripes]	7244	84,666	59,223	57,792	0	2.25872
Contig_1784	Calcineurin subunit B isoform 2 [Lepeophtheirus salmonis]	3.39160	3.67662	6.15579	5.24253	6.49306	4.22602
Contig_1839	Guanine nucleotide-binding protein G(t) subunit α-2-like isoform 2 [Ailuropoda melanoleuca]	3.94329	6.49527	6.90592	6.88416	11.62929	12.47630
Contig_19045	Hypothetical protein TcasGA2_TC003096 [Tribolium castaneum]	2,727	8144	51,268	27,289	72,103	0
Contig_19440	Metabotropic glutamate receptor 5 [Zootermopsis nevadensis]	56,157	47,924	37,713	76,948	26,519	1.43834
Contig_19763	Protein kinase DC2 [Tribolium castaneum]	73,233	80,204	0	81,441	64,555	5.25199
Contig_20075	Glutamate receptor [Culex quinquefasciatus]	0	0	0	11,659	23,105	0
Contig_20180	Phospholipase D1 [Crassostrea gigas]	1.93934	2.43897	1.91928	85,132	1.34962	3.66003
Contig_20395	Ca(2+)/calmodulin-responsive adenylate cyclase isoform X7 [Tribolium castaneum]	0	0	0	0	0	0
Contig_21312	Metabotropic glutamate receptor [Microplitis demolitor]	0	0	0	76,728	0	0
Contig_2172	Hypothetical protein CAPTEDRAFT_204960 [Capitella teleta]	514	15,352	0	4287	0	0
Contig_22568	Adenylate cyclase type 9 isoform X2 [Nasonia vitripennis]	1.01317	75,655	0	1.05630	83,729	4.54126
Contig_22569	Adenylate cyclase type 9 isoform X2 [Nasonia vitripennis]	75,854	90,627	2.85266	7592	0	0
Contig_2322	Serine/threonine-protein phosphatase 2B catalytic subunit 2-like isoform X4 [Nasonia vitripennis]	11.41961	12.93786	5.55333	14.77952	17.57277	22.94517

Table S1. *Cont.*

Contig Number	Blast Result	Male	Male + DM	Male + AZA	Female	Female + DM	Female + AZA
Contig_23307	Phospholipase D delta-like [Glycine max]	3.60308	5.63721	3.22622	3.14827	3.40298	6.15233
Contig_23547	Phospholipase D1-like [Musca domestica]	29,293	0	0	0	0	0
Contig_24026	Voltage-dependent calcium channel type A subunit α-1, partial [Microplitis demolitor]	0	0	0	0	0	0
Contig_25377	Guanine nucleotide-binding protein Gq subunit alpha [Lepeophtheirus salmonis]	1.31286	87,141	1.37147	730	1.44661	0
Contig_25416	Glutamate [NMDA] receptor subunit epsilon-2 [Cerapachys biroi]	6442	0	0	21,491	0	0
Contig_25697	metabotropic glutamate receptor 4, 6, 7, putative [Ixodes scapularis]	0	0	0	13,913	0	0
Contig_26728	Glutamine synthetase 2 [Pacifastacus leniusculus]	68,197	12.22177	0	0	7.51447	0
Contig_26986	Glutamate [NMDA] receptor subunit 1-like [Bombus impatiens]	47,862	26,804	0	1497	39,553	1.07264
Contig_27491	Glutamate receptor ionotropic, kainate 2-like [Bombyx mori]	16,837	0	0	0	0	0
Contig_28168	Voltage-dependent calcium channel type A subunit α-1, partial [Cerapachys biroi]	0	0	0	0	0	0
Contig_28882	Adenylate cyclase type 8 isoform X4 [Acyrthosiphon pisum]	30,238	52,289	29,925	18,583	10,522	0
Contig_288	cAMP-dependent protein kinase catalytic subunit [Diaphorina citri]	14.46456	16.68881	30.43871	10.27855	17.60673	24.34183
Contig_29552	Glutamate receptor, ionotropic kainate 1, 2, 3 (glur5, glur6, glur7) [Aedes aegypti]	0	50,431	0	39,431	66,975	1.21086
Contig_29687	Adenylate cyclase type 2-like, partial [Tribolium castaneum]	0	0	0	36,316	0	0
Contig_30364	Vesicular glutamate transporter 2 [Zootermopsis nevadensis]	19,852	2,9647	4666	8279	16,406	1.77960
Contig_30498	Hypothetical protein DAPPUDRAFT_107323 [Daphnia pulex]	0	0	0	6031	0	0
Contig_30886	Hypothetical protein SINV_02864 [Solenopsis invicta]	0	0	0	11,405	0	0
Contig_31398	Metabotropic glutamate receptor 4-like isoform X4 [Apis dorsata]	0	0	0	16,478	0	0
Contig_33500	GH14412p [Drosophila melanogaster]	1.33944	6.40116	0	44,687	10.62641	0
Contig_33909	Unnamed protein product [Oikopleura dioica]	1.37260	0	0	1.14483	0	0
Contig_33977	Glutamine synthetase [Fenneropenaeus chinensis]	0	0	0	0	0	0
Contig_34161	NMDA-type glutamate receptor subunit 1, variant 5 (NR1.5) [Apis mellifera carnica]	0	2763	0	46,293	0	0
Contig_3442	Guanine nucleotide-binding protein, putative [Ixodes scapularis]	6.57687	5.08772	12.45580	8.83989	7.82038	11.87646
Contig_34712	Hypothetical protein TcasGA2_TC004727 [Tribolium castaneum]	0	0	0	23,072	0	0
Contig_352	Glutamine synthetase 2 [Pacifastacus leniusculus]	180.98730	101.93189	82.24071	159.31614	49.17603	41.60826
Contig_35512	Glutamine synthetase 2 [Acyrthosiphon pisum]	0	0	0	0	0	0
Contig_3556	Predicted protein [Physcomitrella patens]	9.35734	10.10119	20.08136	12.25820	11.26322	19.14734
Contig_3585	Phospholipase D Δ-like [Glycine max]	89,148	0	0	24,785	9823	0
Contig_3586	Phospholipase D delta isoform [Medicago truncatula]	7.56449	8.72055	9.98168	6.86263	7.89642	0
Contig_40177	Phospholipase C, β isoform [Daphnia pulex]	14,647	0	0	8144	0	0
Contig_4025	Solute carrier family 25 member 38 [Caligus clemensi]	29.26067	43.83647	53.69248	23.20139	35.92922	17.61554
Contig_4077	1-phosphatidylinositol 4,5-bisphosphate phosphodiesterase isoform X2 [Acyrthosiphon pisum]	2.32209	3.81303	2.69016	4.14886	6.40268	8.68167
Contig_41892	Glutamate receptor ionotropic, kainate 4 [Alligator sinensis]	83,955	0	0	0	0	0
Contig_4318	Hypothetical protein SINV_16547 [Solenopsis invicta]	3.92327	3.85570	4.28350	4.13778	4.18350	3.63046
Contig_43726	Glutamate receptor ionotropic, NMDA 2B isoform X3 [Acyrthosiphon pisum]	0	0	0	0	0	0
Contig_46561	NMDA receptor subunit 2 variant A4 [Diploptera punctata]	0	0	0	0	0	0
Contig_47494	Hypothetical protein DAPPUDRAFT_309629 [Daphnia pulex]	20,887	20,796	0	0	0	0

Table S1. *Cont.*

Contig Number	Blast Result	Male	Male + DM	Male + AZA	Female	Female + DM	Female + AZA
Contig_47862	Glutamine synthetase 2 cytoplasmic, putative [Pediculus humanus corporis]	25,285	3.02090	0	84,356	0	0
Contig_54132	Conserved hypothetical protein [Pediculus humanus corporis]	0	0	0	0	0	0
Contig_5421	Guanine nucleotide-binding protein G(i) subunit α [Acyrthosiphon pisum]	4.28786	17.29940	6.61863	16.54711	23.69394	32.12763
Contig_543	G protein-coupled receptor kinase 1, partial [Zootermopsis nevadensis]	4.03330	4.81878	9.65240	6.27946	4.84821	5.25912
Contig_54773	Hypothetical protein YQE_09980 partial [Dendroctonus ponderosae]	2.21242	94403	0	3.16334	0	0
Contig_5647	Calcineurin subunit B isoform 1 [Caligus rogercresseyi]	4.01938	5.71684	3.59897	13.24988	10.12308	13.72632
Contig_5661	Phosphatidylcholine-hydrolyzing phospholipase D1	9.89742	10.25955	12.65123	6.52720	12.40792	8.25350
Contig_56735	Muscle calcium channel subunit alpha-1 isoform X1 [Microplitis demolitor]	33,208	0	0	0	0	0
Contig_5765	Excitatory amino acid transporter-like, partial [Bombus impatiens]	23.79100	34.03430	23.54498	17.96313	28.14629	15.71490
Contig_5824	Guanine nucleotide-binding protein G(q) subunit alpha [Microplitis demolitor]	5.28452	9.85316	8.13008	7.18569	10.79879	17.80075
Contig_5829	Hypothetical protein L798_01415 partial [Zootermopsis nevadensis]	2.01006	1.51820	1.30332	2.04263	3.36043	3.31386
Contig_59054	Glutamate receptor, metabotropic [Daphnia pulex]	10,551	0	0	0	0	0
Contig_6064	LOW QUALITY PROTEIN: phospholipase D1-like [Bombus terrestris]	1.31527	1.81317	3.80488	1.60332	1.33778	90,698
Contig_63199	Voltage-dependent p/q type calcium channel [Anopheles darlingi]	0	0	0	0	0	0
Contig_63850	Muscle calcium channel subunit α-1-like [Musca domestica]	34,479	0	0	57,515	0	0
Contig_66323	Voltage-dependent calcium channel type D subunit α-1-like isoform X13 [Ceratitis capitata]	0	0	0	0	0	0
Contig_67316	Hypothetical protein SINV_02864 [Solenopsis invicta]	0	0	0	0	0	0
Contig_68789	Glutamate receptor ionotropic, NMDA 2B-like isoform X5 [Nasonia vitripennis]	0	0	0	0	0	0
Contig_68935	Putative MAPK [Oxytricha trifallax]	0	0	0	0	0	0
Contig_69743	Protein kinase domain containing protein [Oxytricha trifallax]	0	0	0	0	0	0
Contig_70030	Ionotropic glutamate receptor subunit GluR4 [Xenopus laevis]	0	0	0	28,218	0	0
Contig_7211	Hypothetical protein DAPPUDRAFT_56427 [Daphnia pulex]	2.30229	3.43832	0	1.66420	1.52210	0
Contig_72263	cAMP protein kinase [Phytophthora infestans T30-4]	0	0	0	0	0	0
Contig_72276	Protein kinase domain protein [Ichthyophthirius multifiliis]	0	0	0	0	0	0
Contig_7291	Mitogen-activated protein kinase 3 [Lepeophtheirus salmonis]	1.66927	2.53521	2.12801	1.27428	5.05036	3.04356
Contig_73721	Hypothetical protein GLOINDRAFT_336006 [Rhizophagus irregularis DAOM 181602]	0	0	0	0	0	0
Contig_74161	Glutamine synthetase [Oxytricha trifallax]	0	0	0	0	0	0
Contig_74544	AGC/PKA protein kinase [Saprolegnia parasitica CBS 223.65]	0	0	0	0	0	0
Contig_74729	Glutamate receptor ionotropic, kainate 2-like isoform X1 [Apis dorsata]	0	0	0	0	0	0
Contig_75638	Protein kinase domain containing protein [Tetrahymena thermophila]	0	0	0	0	0	0
Contig_75806	Phospholipase D1 [Dictyostelium fasciculatum]	0	0	0	0	0	0
Contig_7666	Homer, putative [Pediculus humanus corporis]	1.38839	4.01316	3.36859	5.08021	6.81019	8.02977
Contig_76707	Phospholipase D1, partial [Trichuris trichiura]	0	0	0	0	0	0
Contig_77737	CMGC/MAPK protein kinase, variant 1 [Aphanomyces astaci]	0	0	0	0	0	0
Contig_78491	Hypothetical protein AURANDRAFT_71821 [Aureococcus anophagefferens]	0	0	0	0	0	0
Contig_79849	AGC/PKA protein kinase [Saprolegnia parasitica CBS 223.65]	0	0	0	0	0	0
Contig_79857	PHOSPHOLIPASE D BETA 1 family protein [Populus trichocarpa]	0	0	0	0	0	0
Contig_80293	Hypothetical protein [Paramecium tetraurelia strain d4-2]	0	0	0	0	0	0

Table S1. *Cont.*

Contig Number	Blast Result	Male	Male + DM	Male + AZA	Female	Female + DM	Female + AZA
Contig_80368	Protein kinase domain-containing protein [Euplotes aediculatus]	0	0	0	0	0	0
Contig_82254	MAPK-related kinase [Tetrahymena pyriformis]	0	0	0	0	0	0
Contig_82786	Hypothetical protein [Paramecium tetraurelia strain d4-2]	0	0	0	0	0	0
Contig_9100	Excitatory amino acid transporter 3 [Lepeophtheirus salmonis]	4.80411	8.16423	3.89372	4.07598	5.20226	0
Contig_9379	Phospholipase D α 2-like [Cucumis sativus]	14,587	14,524	45,716	48,667	64,294	0
Contig_9556	Glutaminase liver isoform, mitochondrial [Nasonia vitripennis]	2.21817	1.89297	2.70840	31,235	28,568	1.03297
Contig_9750	Ca(2+)/calmodulin-responsive adenylate cyclase isoform X6 [Tribolium castaneum]	1.22851	48,281	3.64741	1.40214	1.06868	2.31851
